# A Delphi Consensus on Optimising the Care Pathway for Adult Patients With Acute Myeloid Leukaemia (AML): Strategies to Enhance Transplant Accessibility and Feasibility in the United Kingdom

**DOI:** 10.1002/jha2.70353

**Published:** 2026-07-13

**Authors:** Priyanka Mehta, Francesca A. M. Kinsella, Thomas P. Coats, Anjum B. Khan, Anne‐Louise Latif, Victoria T. Potter

**Affiliations:** ^1^ University Hospitals of Bristol and Weston NHS Trusts Bristol UK; ^2^ Birmingham Centre for Cellular Therapy and Transplantation University Hospitals Birmingham Birmingham UK; ^3^ Haematology Department Royal Devon and Exeter University Hospital Exeter UK; ^4^ Department of Haematology Leeds Teaching Hospitals NHS Trust Leeds UK; ^5^ Department of Haematology and Cellular Therapy Queen Elizabeth University Hospital Glasgow UK; ^6^ Department of Haematological Medicine Kings College Hospital NHS Foundation Trust London UK

**Keywords:** consensus, myeloid leukaemia, transplants

## Abstract

**Background:**

Allogeneic haematopoietic stem cell transplant (allo‐HSCT) is a curative option for a large proportion of acute myeloid leukaemia (AML) patients, but availability remains challenging and transplantation is underutilised in the United Kingdom. This consensus aimed to define an optimal UK care pathway for AML and develop recommendations to improve transplant accessibility.

**Methods:**

A steering group of six clinicians, experienced in AML management and stem cell transplantation, designed 47 consensus statements around the optimal UK transplant pathway. These were distributed as a survey to a broader group of clinicians involved in AML management. The threshold for consensus was set at 75% agreement.

**Results:**

A total of 75 responses were received, including 60 haematologists and 15 transplant specialists. All statements reached consensus. Results emphasise the need to involve transplant centres promptly, initiate donor searches early, properly document treatment decisions, and define communication protocols between centres and patients. The findings informed a set of recommendations for care and a clinical checklist.

**Conclusion:**

There is clear willingness to shift the AML treatment paradigm and improve accessibility of allo‐HSCT. The proposed recommendations focus on improving time to transplant, patient assessment and communication protocols. Alongside the checklist, they offer a practical framework to improve services.

Trial Registration: The authors have confirmed clinical trial registration is not needed for this submission

## 1 Introduction

Acute myeloid leukaemia (AML) is a haematopoietic stem cell malignancy characterised by the clonal expansion of immature myeloid blasts in the bone marrow and peripheral blood [1]. Although AML can occur at any age, it is most prevalent in adults, with incidence increasing with advancing age; the median age at diagnosis is around 70 years [2, 3]. In the United Kingdom, approximately 2700 new cases are diagnosed annually, and overall outcomes remain poor, with 5‐year survival rates of only 13.6% [4]. AML also imposes substantial morbidity, prolonged hospitalisations and significant adverse impact on quality of life [5, 6].

The treatment landscape for AML has progressed over the past decade, driven by advances in molecular diagnostics, measurable residual disease (MRD) assessment and targeted therapies [7]. Therapy selection is dependent on patient characteristics, such as performance status, comorbidities and underlying disease biology. The mainstay of treatment in the UK is induction therapy, either at high or low intensity, followed by consolidation therapy to sustain remission [8]. For most people in good general health and with intermediate or high‐risk disease, an allogeneic haematopoietic stem cell transplant (allo‐HSCT) remains the only curative therapy [1, 8].

Despite its powerful therapeutic potential, only a minority of patients proceed to allo‐HSCT, with only 674 stem cell transplants carried out for AML in the UK in 2024 [9]. Real‐world data suggest the United Kingdom has amongst the lowest transplant rates in Europe at only 34%, compared to France (36%) and Germany (62%), despite having similarly aged patient populations. This highlights inequities in transplant access and outcomes stemming from structural differences in referral patterns and treatment decisions [10, 11, 12]. Barriers within the UK include access to transplant services (based on geographical distance to centres), frailty and/or comorbidities, patient or physician preference and socioeconomic factors [13, 14]. The 2022 European LeukemiaNet (ELN) recommendations provide guidance on the indications for allo‐HSCT, including patient eligibility [14]. However, in the United Kingdom, AML management varies between centres, partly due to the absence of a nationally standardised framework for assessing transplant eligibility [8]. Regional disparities in service delivery further contribute to inconsistent access and outcomes. The ‘hub‐and‐spoke’ model used by several UK regions links specialist transplant centres with hospitals to provide opportunities for expert transplant advice and optimise the management of AML [8]. This model relies heavily on effective coordination and communication between hospitals and transplant centres; if this is suboptimal, there can be delays in donor identification and transplantation.

The ELN recommendations emphasise the importance of initiating donor searches at diagnosis for patients likely to require allo‐HSCT [14]. In the United Kingdom, however, transplantation is discussed variably for intermediate or high‐risk disease through or after the patient's induction chemotherapy, or when patients are refractory or have relapsed [15, 16]. This reactive approach may lead to missed opportunities for timely donor searches, as patients can relapse or become unfit for transplantation due to prolonged treatment. UK studies have indicated that many patients undergo additional ‘holding’ or consolidation cycles of chemotherapy due to a delay in transplant.

Overall, allo‐HSCT remains underutilised, highlighting the need for earlier referral and improved coordination of transplant planning [17, 18]. This modified Delphi study aimed to establish expert consensus on the optimal care pathway for patients with AML in the United Kingdom and provide recommendations to improve transplant accessibility and streamline care.

## 2 Methods

**FIGURE 1:**
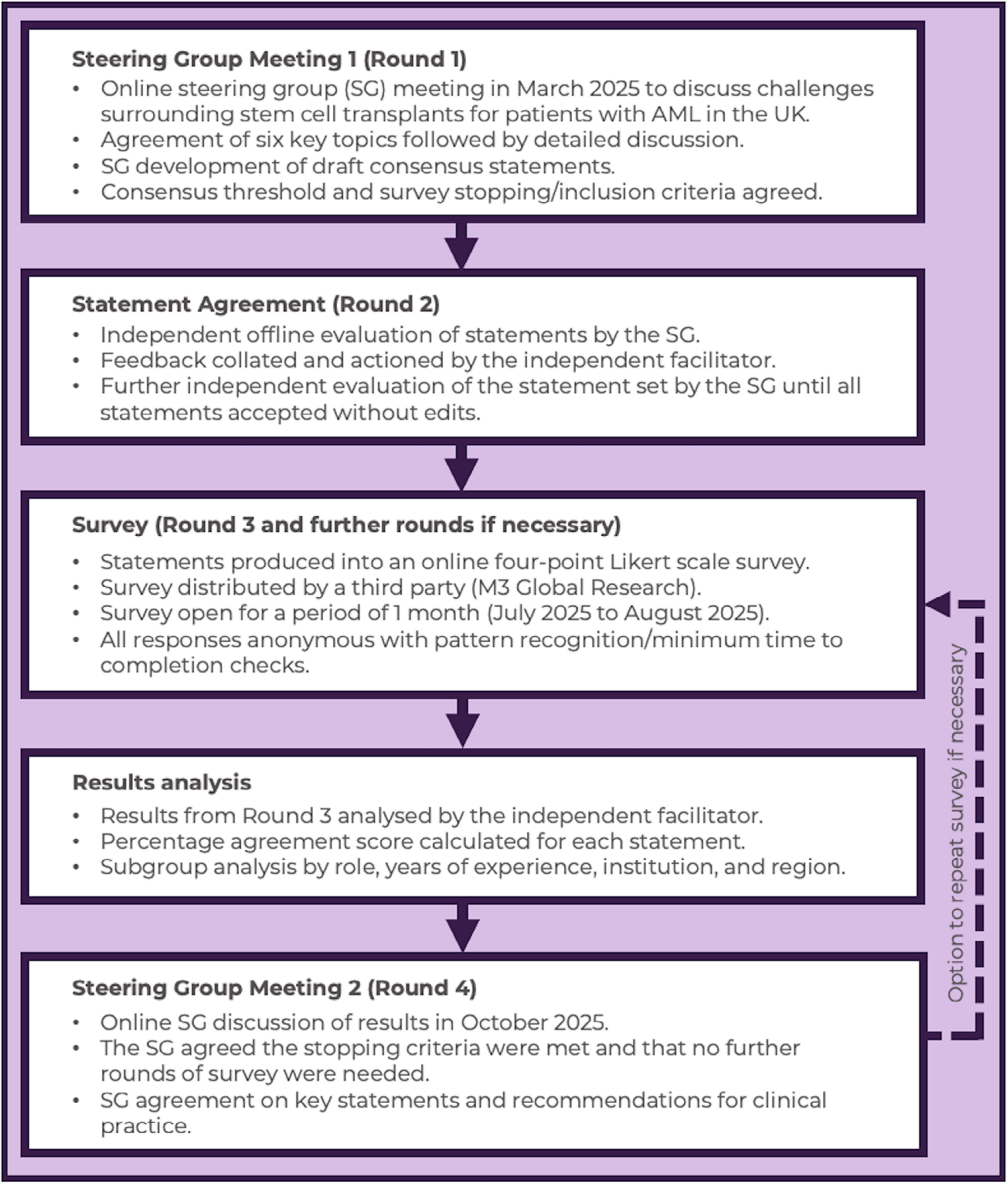
Modified Delphi study design.

The process followed a modified Delphi methodology (Figure 1), guided by an experienced independent Delphi facilitator (Triducive Partners Limited). The study protocol was not registered. All information is reported following the ACCORD guidelines [19].

In February 2025, a literature review was conducted examining transplant suitability, treatment access and pathway management for AML in the United Kingdom. The search was conducted using publicly available resources, including PubMed and Google Scholar. The review encompassed material published within the last 10 years, with specific focus on those published within the last 5 years. Search terms included, but were not limited to, ‘AML transplant’, ‘AML pathway’ and ‘AML challenges’. Findings from the review were used to develop the scope of the research and define questions for the initial steering group (SG) meeting.

### 2.1 SG Meeting 1 (Round 1)

A SG of six UK clinicians (six haematologists, including two transplant specialists) convened virtually in March 2025. The SG was selected based on their clinical experience managing AML and involvement in stem cell transplantation, their research background and their publication history. The group discussed factors affecting optimal transplant uptake in the United Kingdom, transplant eligibility and optimal referral pathways. Six topics of focus for the research were identified:

1. Early transplant discussion
2. Patient suitability
3. Time to transplant
4. The role of MDT assessment in transplant referral
5. Standardising the referral process and communication between centres
6. The role of shared care

In alignment with these topics, 53 draft consensus statements were generated. Stopping criteria for the survey were established a priori as a 1‐month window to collect responses, a target of 75 responses, 90% of statements passing the threshold for consensus, and a threshold for consensus set at ≥ 75% (a widely accepted threshold) [20]. These criteria were established in order to gather a spread of opinions, while acknowledging workload pressures in the NHS.

### 2.2 Statement Agreement (Round 2)

Following the meeting, the 53 draft statements were collated by the facilitator and independently rated by the SG as either ‘accept’, ‘remove’, or ‘reword’. Feedback during this stage was qualitative, as per standard Delphi methodology [21], and members were able to propose new statements. The facilitator implemented required changes, and the revised statements were re‐circulated for independent SG review. This process resulted in a final set of 47 statements.

### 2.3 Survey (Round 3)

The finalised statements were developed into an online survey, presenting each statement alongside a 4‐point Likert scale (‘strongly disagree’, ‘tend to disagree’, ‘tend to agree’ and ‘strongly agree’). The survey was distributed by a third party (M3 Global Research) to health care professionals (HCPs) in July 2025 and remained open for 4 weeks, closing in August 2025.

Recruitment of panel members was according to the following criteria (as agreed by the SG):

1. Located in the United Kingdom
2. Working in a relevant role (haematologist or stem cell transplanter)
3. Experienced in managing patients with AML
4. Primarily affiliated with a transplant centre or a hospital providing intensive, supportive, or low‐intensity AML care

Any respondents not meeting the eligibility criteria were excluded. Demographic data (role, years of experience, institution and region) were captured based on the inclusion criteria and for sub‐analyses. A statement of consent was included at the start of the survey, and consent was implied by survey completion. Anonymity was planned in the study design: no personal information or protected characteristics were recorded, and the identities of respondents were not known to either the SG or the facilitator. This study did not require ethical approval, as it was a non‐interventional opinion‐based Delphi process involving HCPs, with no collection of clinical or patient‐related data. All respondents received a nominal fee for completing the survey.

### 2.4 Results Analysis

Survey responses were screened against inclusion criteria and assessed for validity using pattern recognition and a minimum completion time of 2.5 min to support data integrity. Valid surveys were analysed by the independent facilitator to produce an arithmetic agreement score for each statement.

### 2.5 SG Meeting 2 (Round 4)

The SG convened in October 2025 to review and interpret the survey results. The group agreed that the stopping criteria had been met and, while a second round of survey had been anticipated, due to the high consensus, no further rounds were necessary. Key statements were identified for each topic, and draft recommendations were developed based on the consensus findings. These draft recommendations were then independently reviewed and refined by each SG member as part of the manuscript development process.

## 3 Results

Of the draft 53 statements generated in Round 1, six were removed, 23 were modified, three new statements were added, and 21 were agreed upon for inclusion without modification. This led to a final set of 47 statements for survey testing.

A total of 75 responses to the survey were received and included in the final analysis. Respondents were predominantly haematologists (*n* = 60, 80%), with the remainder being stem cell transplant clinicians (*n* = 15, 20%) (Figure S1). Respondents predominantly had ≥ 5 years of experience in their role (*n* = 72, 96%), with more than half reporting 11 or more years in their role, and representation varied across institution types (Figures S2 and S3). Responses were received from across the United Kingdom, with London having the greatest representation (*n* = 19, 25%) (Figure S4)

**FIGURE 2:**
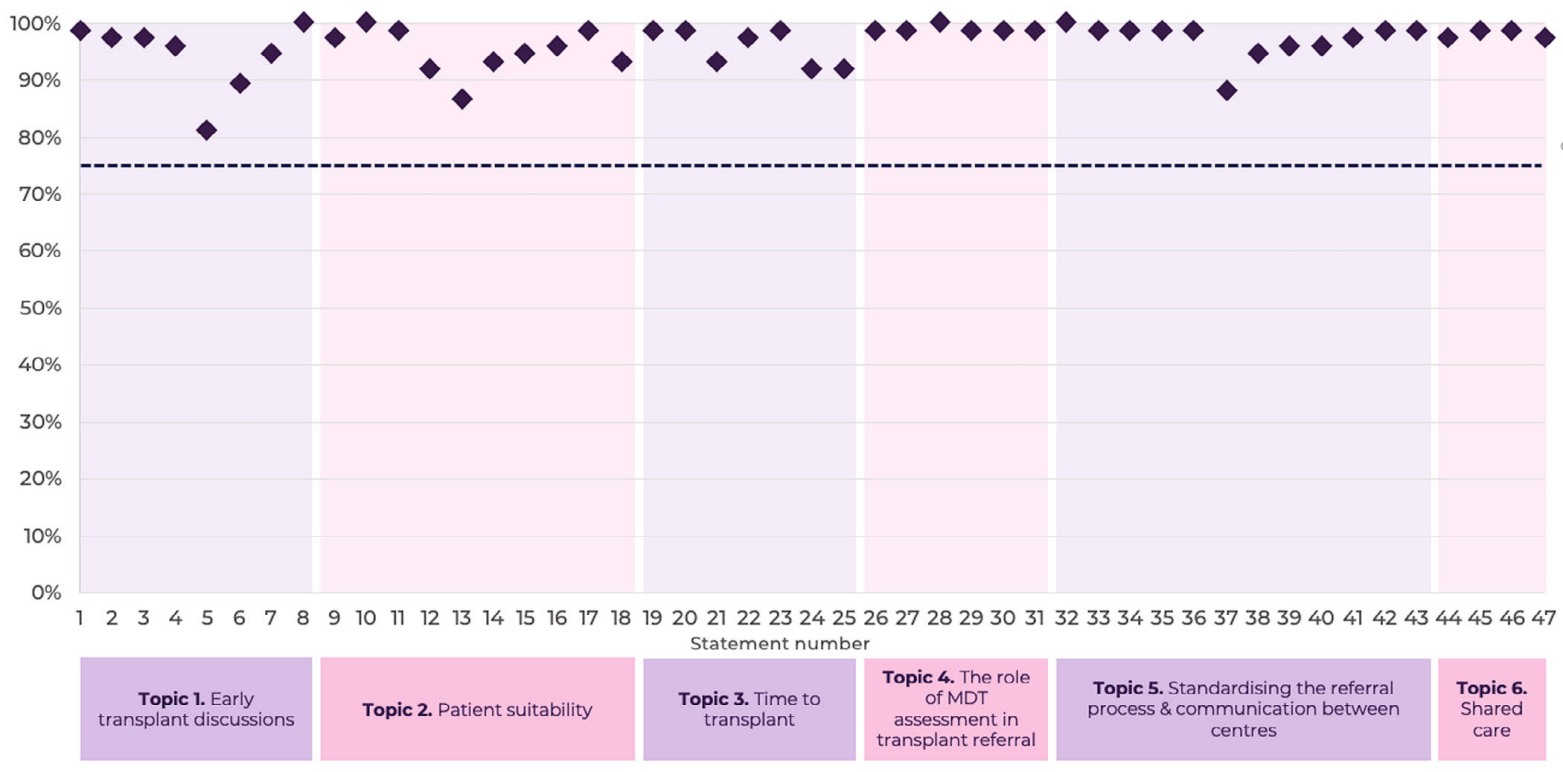
Consensus agreement levels by statement. The threshold for consensus is depicted by the dashed line (≥ 75%).

Consensus was achieved for all 47 statements (100%), with all but four reaching ≥ 90% agreement. Statements and corresponding levels of agreement are presented in Table 1 and illustrated in Figure 2. As the predefined stopping criteria were met, no additional survey rounds were required.

**TABLE 1:** Defined consensus statements and corresponding levels of agreement. All values are rounded to the nearest number.

**No**.	**Statement**	**Strongly agree**	**Tend to agree**	**Tend to disagree**	**Strongly disagree**	**Agreement score** **^a^** **(*n* = 75)**
**Topic 1. Early transplant discussion**						
**1**.	All patients diagnosed with acute myeloid leukaemia (AML) and eligible for curative treatment should be informed at diagnosis that stem cell transplantation may be part of their treatment pathway	80%	19%	1%	0%	**99%**
**2**.	All patients diagnosed with AML who have the potential for curative treatment (excluding acute promyelocytic leukaemia [APL]) should undergo human leukocyte antigen (HLA) typing at diagnosis to facilitate timely donor identification	80%	17%	1%	1%	**97%**
**3**.	Donor searches should be initiated as early as possible and suitable related donor options should be explored promptly with the patient at diagnosis	72%	25%	1%	1%	**97%**
**4**.	Transplant centres should be involved in the donor search to allow prompt initiation of an unrelated donor search should no suitable related donor is identified	69%	27%	3%	1%	**96%**
**5**.	There is lack of evidence supporting an age cut‐off for suitable sibling donors	17%	64%	19%	0%	**81%**
**6**.	To speed up donor workup, an age cutoff for sibling donors should be set by transplant centres to make pathways for referring hospitals clearer	39%	51%	9%	1%	**89%**
**7**.	All patients diagnosed with AML who have the potential for curative treatment (excluding APL) should be referred to a transplant specialist as soon as possible, within their first treatment cycle and molecular results sent as they become available	64%	31%	5%	0%	**95%**
**8**.	Measurable residual disease (MRD) markers should be identified at diagnosis, and subsequently monitored in accordance with ELN recommendations to support transplant decision‐making	77%	23%	0%	0%	**100%**
**Topic 2. Patient suitability**						
**9**.	Transplant recommendations should balance the risk of disease progression without transplant against the risks and toxicity associated with transplantation	72%	25%	3%	0%	**97%**
**10**.	All patients should undergo a comprehensive assessment of performance status, comorbidities and organ function, such as Haematopoietic Cell Transplantation‐specific Comorbidity Index (HCT‐CI) scoring, to evaluate their transplant risk	85%	15%	0%	0%	**100%**
**11**.	Fitness for transplant should be assessed holistically, including evaluation by a clinical psychologist, physiotherapist and dietitian as part of the multidisciplinary team (MDT)	60%	39%	1%	0%	**99%**
**12**.	Age alone should not be a barrier to transplant	53%	39%	8%	0%	**92%**
**13**.	Patients over 60 should consider undergoing a formal geriatric assessment	45%	41%	13%	0%	**87%**
**14**.	To improve standardisation of care and referral consistency, there should be strict disease criteria for transplant indications, as there are for other Cancer drug fund/NICE approved treatments, for example, *TP53* mutated disease, MRD‐positive disease, transplant in partial remission	59%	35%	7%	0%	**93%**
**15**.	Patients with primary refractory AML should be considered for transplant	64%	31%	5%	0%	**95%**
**16**.	Patient fitness for transplant should be reassessed after each cycle of chemotherapy	65%	31%	4%	0%	**96%**
**17**.	MRD positivity should not be a barrier to transplant but the patient may require additional therapy, especially if there is a time delay in securing a donor/transplant slot	44%	55%	1%	0%	**99%**
**18**.	MRD‐positive patients should undergo a higher intensity conditioning, such as myeloablative conditioning, before transplant if possible	37%	56%	5%	1%	**93%**
**Topic 3. Time to transplant**						
**19**.	Transplant delays can be reduced through early referral to a transplant centre	73%	25%	1%	0%	**99%**
**20**.	Early donor search and HLA typing help to prevent transplant delays	79%	20%	1%	0%	**99%**
**21**.	All eligible patients in first complete remission (CR1) should proceed to transplant as soon as adequate disease control is achieved and a suitable donor is available	68%	25%	7%	0%	**93%**
**22**.	All eligible patients in second complete remission (CR2) should proceed to transplant as soon as adequate disease control is achieved and a suitable donor is available, if they did not have a transplant in CR1	69%	28%	3%	0%	**97%**
**23**.	Patients should have comorbidities optimally managed during induction therapy to minimise transplant‐related risks	72%	27%	1%	0%	**99%**
**24**.	Additional chemotherapy cycles before transplant should be minimised once a patient has achieved CR1 unless necessary for disease control or relapse prevention	56%	36%	8%	0%	**92%**
**25**.	If additional chemotherapy cycles are administered after adequate disease control but before transplant, the MDT should investigate the reasons why this happened	45%	47%	8%	0%	**92%**
**Topic 4. The role of MDT assessment in transplant referral**						
**26**.	All AML patients for whom transplant is an option should be discussed in a transplant MDT	79%	20%	1%	0%	**99%**
**27**.	MDT discussions should include at least two clinicians with a special interest in AML, and should also involve input from a transplant specialist or transplant centre; otherwise, the patient should be referred to a regional AML MDT or an AML/transplant specialist at another centre	79%	20%	1%	0%	**99%**
**28**.	All AML clinicians should have access to a regional AML MDT to discuss complex patient cases	88%	12%	0%	0%	**100%**
**29**.	The reasons behind decisions to refer or not refer an AML patient for transplant should be formally documented at diagnosis during an MDT meeting	80%	19%	1%	0%	**99%**
**30**.	All transplant‐eligible AML patients should be discussed at an MDT meeting and with the transplant team when an integrated genetic (Haematological Malignancy Diagnostic Service, HMDS) report becomes available	72%	27%	1%	0%	**99%**
**31**.	A transplant centre should provide input into patient management during MDT discussions	72%	27%	1%	0%	**99%**
**Topic 5. Standardising the referral process and communication between centres**						
**32**.	Every referring hospital should have a designated transplant centre for AML referrals and discussions	80%	20%	0%	0%	**100%**
**33**.	Transplant centres should provide clear, standardised referral guidelines and processes for referring hospitals	79%	20%	1%	0%	**99%**
**34**.	MDT proformas should be standardised to ensure consistency and clarity in treatment decisions	80%	19%	1%	0%	**99%**
**35**.	Transplant centres and referring hospitals should establish clear communication protocols, including designated key contacts (e.g., clinical nurse specialists [CNS]) and a shared email address for the transplant centre	73%	25%	1%	0%	**99%**
**36**.	Efforts should be made to develop an electronic platform to enhance communication between transplant centres and referring hospitals	71%	28%	1%	0%	**99%**
**37**.	All transplant eligible patients should receive written information about the transplant process at diagnosis	55%	33%	12%	0%	**88%**
**38**.	All transplant eligible patients should receive written information about the transplant process when they achieve CR1 after first cycle of chemotherapy	63%	32%	5%	0%	**95%**
**39**.	The transplant and treating centres should agree on key patient outcomes that must be shared to ensure both teams are fully informed prior to the transplant	65%	31%	4%	0%	**96%**
**40**.	After each treatment cycle, disease reassessment, fitness reassessment and adverse events should be communicated to the transplant centre	67%	29%	4%	0%	**96%**
**41**.	Transplant centres should provide provisional transplant dates to the referring team to allow timely treatment and testing	68%	29%	3%	0%	**97%**
**42**.	Transplant decisions must be clearly communicated to the referring team by the transplant centre	84%	15%	1%	0%	**99%**
**43**.	Predicted outcomes, including transplant‐related mortality (TRM), overall survival and relapse risk, should be clearly communicated to the referring team and the patient (if the patient consents)	77%	21%	1%	0%	**99%**
**Topic 6. The role of shared care**						
**44**.	The transplant centre should define the post‐transplant follow‐up schedule before the transplant, including the frequency and location of reviews and monitoring	64%	33%	3%	0%	**97%**
**45**.	Specific shared care responsibilities, such as local blood tests, transfusion support and management of complications, should be agreed upon between the transplant centre and referring hospitals before transplantation	71%	28%	1%	0%	**99%**
**46**.	The agreed shared care plan, including responsibilities and follow‐up schedules, should be clearly explained to the patient and carers before transplantation	81%	17%	1%	0%	**99%**
**47**.	Shared care forms should include detailed patient clinical information and be communicated to local blood banks	72%	25%	3%	0%	**97%**

^a^ Percentages were rounded to the nearest whole number prior to assessing whether the threshold for consensus was met.

Sub‐analyses were performed to examine the consensus by role, years of experience, institution and region. Results were analysed for variation in responses > 10% more or less than the mean agreement, or with at least one role not achieving the threshold of ≥ 75%. The results from these analyses are presented in Tables S1–S4. Following review of the findings, the SG agreed on nine recommendations to inform the transplant process for adult patients with AML. A clinical checklist is also presented as Supporting Information to help apply these recommendations in routine care.

## 4 Discussion

### 4.1 Recommendations

Consensus across all statements showed overwhelming agreement with the need to reduce the time to transplant for eligible patients, as well as with strategies to help streamline processes to enable more timely access to allo‐HSCT. Based on the findings, the SG developed the recommendations for optimising AML care listed below:

1. **Early transplant discussion**: At the time of diagnosis, all patients with AML who are eligible for curative treatments should be informed that stem cell transplantation may be part of their treatment pathway and should be referred promptly to a transplant specialist to initiate donor searches.
2. **Comprehensive assessment**: Transplant recommendations must balance the risk of disease progression without a transplant against the toxicity associated with transplantation, plus the risk of subsequent relapse. Assessment should incorporate:
  - Patient factors such as performance status, comorbidities and organ function.
  - Disease factors such as TP53 mutation, MRD positivity, partial remission status and refractory disease (if treatment sensitive).
3. **Age considerations**: Age alone should not be a barrier to transplant, and patients over 60 should be considered for geriatric assessments to support appropriate treatment decision‐making.
4. **Integration of genetic/cytogenetic findings**: All AML patients should be discussed at a multidisciplinary team (MDT) meeting with the transplant team once the integrated genetic (Haematological Malignancy Diagnostic Service, HMDS) report becomes available, with formal documentation of the decision and its rationale.
5. **Regional MDT review**: All clinicians should have access to regional MDTs (which include at least two AML experts and a transplant specialist) to discuss complex cases, and all treatment decisions should have their rationale formally documented.
6. **Timing of transplant**: Eligible patients in first complete remission (CR1) should proceed to transplant as soon as disease control, patient fitness and a suitable donor are confirmed. If that is not feasible, transplant should occur in second remission (CR2) under the same principles.
7. **Delays and accountability**: When additional cycles of chemotherapy are administered after adequate disease control has been achieved (i.e., a delay in transplant), the MDT should review and document the reasons for delay to identify and address potential system or process barriers.
8. **Post‐transplant planning**: The transplant centre should define and communicate the post‐transplant follow‐up schedule, including the frequency and location of reviews and monitoring, before transplantation occurs.
9. **Communication protocols**: To streamline services and ease communication between patients, referrers and transplant centres, communication protocols must specify:
  - The point of contact from the referral team.
  - The point of contact from the transplant centre.
  - The intended patient outcomes.
  - The results of any disease and fitness assessments conducted throughout the process.
  - Written information on the transplant process and the patient's predicted outcomes, including transplant‐related mortality (TRM), overall survival and relapse risk.

### 4.2 Transplant Timing and Eligibility

Allo‐HSCT is still the only curative treatment for a significant proportion of patients with AML [1, 8], and reducing the time to transplant was a key theme across the statements. Within Topic 1, respondents supported the need for early discussions of transplantation therapy with patients and for prompt engagement of transplant centres. Early HLA testing and CR1 transplantation lead to improved overall survival [22], but there can be challenges identifying appropriately matched donors, particularly those in ethnic minority groups [23]. Recent data support the concept of early tissue typing in patients diagnosed with AML, especially in ethnic minority patients, who benefit from optimised donor search strategies and expedited molecular testing to reduce delay and improve risk stratification [24]. Yet UK‐based research shows many patients receive third and fourth cycles of chemotherapy consolidation having already achieved CR1 due to delays in transplant, and that allo‐HSCT is underused [17, 18]. These factors underpin the need to engage with transplant centres early and initiate donor searches promptly to minimise the time to transplant and ensure transplants occur in CR1 where indicated. Information on both matched and mismatched donors (including mismatched unrelated haploidentical and cord blood) should be gathered in a timely fashion to ensure backup donors are in place if plans for the first‐choice donor fall through. Following the advent of post‐transplant cyclophosphamide as effective graft‐versus‐host‐disease prophylaxis in the setting of mismatched donors [25], UK searches can be broadened to include HLA‐mismatched donors from the outset, potentially expediting donor identification [26]. This issue is particularly important as successful use of mismatched donors for patients lacking a matched donor increases and outcomes appear equivalent to those with matched donors [25, 27]. Notably, a prospective study conducted by the Blood and Marrow Transplant Clinical Trial Network utilised a rapid alternative donor search strategy and demonstrated equivalent time to transplant between those very likely and very unlikely to find a matched donor [28].

Transplant eligibility is complex and multifactorial, requiring consideration of both patient and disease factors [29, 30]. In line with the literature [31, 32], agreement in Topic 2 shows that age alone should not be a barrier to transplant. However, patients over 60 years old may require additional considerations, such as formal geriatric assessments [32] or equivalent fitness testing where this is not available, to help guide personalised treatment choices. Consideration of disease factors, such as cytogenetic risk and MRD status, is necessary for all patients [29]. However, MRD negativity is not a prerequisite for transplant, and those who are MRD‐positive may still benefit from transplant over further chemotherapy alone but could require additional therapy or higher intensity transplant conditioning where possible. MRD sampling and testing should be performed following international guidance [14, 33]. Further work is required to validate use of NGS‐based monitoring for all lesions and establish the required depth of detection [33]. Patients who are MRD‐positive before transplant should also receive post‐transplant maintenance treatment where available and appropriate, along with other post‐transplant modifying approaches to prevent relapse [30, 32, 34]. While not considered within the Delphi, decision support tools based on large data sets can be used to aid clinical decision‐making [14, 35].

### 4.3 Standardising Multidisciplinary Care

Treatment decisions must balance the risk of disease progression without transplant against the toxicity associated with transplantation itself, plus the risk of subsequent disease relapse. It is recommended that cases with complex disease and/or fitness scenarios be discussed in a regional MDT with AML and transplant specialists present. Given the variable access to regional MDTs across the United Kingdom, developing regional groups, while formalising existing forums, will be essential to support equitable access to transplants and improved patient outcomes. NICE guidance sets out the importance of fully integrated HMDS reports, and these should be discussed within MDTs (local or regional) once they are finalised to explore appropriate options for care [36]. It should be noted that HMDS reports are a requirement, and that consistent access is required across the country to services which provide rapid turnaround for the tests which contribute to these reports [36]. It is acknowledged that the range of tests available and turnaround times vary across the United Kingdom. This impacts the delivery of the completed HMDS report and warrants multiple discussions as results become available. Work is currently ongoing across the United Kingdom to assess and address MDT working, including optimising the turnaround time for genetic testing and updating existing recommendations for laboratory testing [37].

Responses from Topics 4 and 5 emphasise a need to streamline AML care to help expedite treatment and demonstrate willingness amongst clinicians to address current bottlenecks within the treatment process. While guidelines exist to help ensure standards and effectiveness of transplant centres and MDTs [36, 38], long‐term follow‐up remains challenging, with recognised implementation barriers. JACIE accreditation outlines the responsibilities of transplant centres throughout treatment and patient follow‐up; however, difficulties in implementing long‐term follow‐up in clinics have been noted [39]. Communication protocols between transplant centres, referrers and patients vary considerably across the UK. While not prescriptive, as each centre/clinic will have different issues that need to be addressed, based on the current consensus, centres should define points of contact from referral teams and transplant centres, standardise referral information and specify data requirements to ensure compliance with JACIE standards. A checklist to help guide the process is presented within the Supporting Information. This utilises the statements from the survey to create a series of steps to follow in order to support communication between services and provide consistency of care to patients.

### 4.4 Strengths and Limitations

The study achieved high levels of consensus from 75 HCPs experienced in AML care from across the United Kingdom, with over 50% of respondents having ≥ 11 years of experience in the role. While the high levels of consensus could indicate acquiescence bias, it is emphasised that the statements were designed to represent best practice, not current practice. Therefore, it is encouraging to see such high levels of agreement on some core aspects of transplant care. The robustness of the responses was ensured using pattern recognition and a minimum time to completion.

Low response rates from Scotland limited the geographic sub‐analyses. As there are differences in the structure of transplant services provided by NHS England and NHS Scotland, future research aiming to compare opinions from service providers in these regions is warranted. While the 4‐point Likert scale is seen as a strength to help avoid middle‐option bias, it is possible that some nuance within the results was lost. However, 16 statements had ≥ 75% of respondents selecting ‘strongly agree’ as opposed to ‘tend to agree’, showing clear, irrefutable consensus for these statements. Finally, care for patients with AML is incredibly complex and clinical decision‐making relies on consideration of an array of nuanced information. This study does not explore variables on a case‐by‐case basis; instead, it provides overarching guidance to help improve communication and transplant decision‐making.

## 5 Conclusion

This modified Delphi exercise achieved strong agreement from a panel of 75 healthcare practitioners from across the United Kingdom on a series of 47 statements outlining best practice in AML care and allo‐HSCT. The high level of agreement amongst respondents emphasises the desire to shift the AML treatment paradigm towards a higher rate of curative transplantation in AML patients by streamlining care and improving communication between referral centres, transplant centres and patients. The findings underscore the critical importance of developing treatment protocols that minimise the time to transplant, reflecting broader literature from AML studies. This consensus exercise enabled the development of a series of actionable recommendations and a clinical checklist which, if implemented, could strengthen communication throughout the treatment pathway and help expedite donor searches and ensure timely progression to transplant.

## Author Contributions

All authors developed the initial statements, contributed equally to the analysis and discussion of results and read and approved the final manuscript.

## Funding

The study was initiated and funded by Jazz Pharmaceuticals. All authors received funding from Jazz Pharmaceuticals while undertaking this study. Jazz Pharmaceuticals commissioned Triducive Partners Limited to facilitate the project and analyse the responses to the consensus statements in line with the Delphi methodology. After engaging Triducive Partners Limited, Jazz Pharmaceuticals made no contribution to the design and development of the study outside of payment of honoraria. Jazz Pharmaceuticals took no part in the writing, revision or editing of the manuscript except to check that the manuscript contained no promotion of specific medicines.

## Ethics Statement

This study did not require registration because neither the assigned interventions nor the outcomes assessed were related to the health of participants. This study did not require ethical approval, as it was a non‐interventional opinion‐based Delphi process involving HCPs, with no collection of sensitive or personally identifiable data, and all responses were anonymised. In accordance with the Governance Arrangements for Research Ethics Committees (GAfREC) Section 2.3, research involving staff recruited by virtue of their professional role does not require Research Ethics Committee review unless it involves access to confidential information or raises issues of professional performance.

## Consent

All participants were provided with information about the study and gave informed consent to participate.

## Conflicts of Interest

All authors received honoraria from Jazz Pharmaceuticals while undertaking this study. Jazz Pharmaceuticals commissioned Triducive Partners Limited to facilitate the project and analyse the responses to the consensus statements in line with the Delphi methodology. Priyanka Mehta has received speaker/advisory fees from AbbVie, Astellas, Daiichi Sankyo, Jazz Pharmaceuticals, Novartis, Otsuka, Servier and Stemline. Francesca A.M. Kinsella has received honoraria from Vertex, Sanofi, Incyte and Therakos, has been on advisory boards for Vertex, Incyte and Sanofi, and has received research funding from Gilead. Thomas P. Coats has received medical education grants from Pfizer and Servier, and consultation fees and conference travel/attendance fees from Astellas and Jazz. Anjum B. Khan has received honoraria from AbbVie, Astellas, Incyte, Immedica, Jazz, Medac, Novartis, Otsuka, Servier, Synairgen and TC BioPharm. Anne‐Louise Latif has received honoraria or consultation fees from AbbVie, Astellas, Kite and Novartis, and taken part in sponsored speakers bureaux for Astellas, Kite and Novartis. The other author declares no conflicts of interest.

## Supporting information




**Supporting Information**: EJH_AML Consensus_Supplementary Clinical Checklist.docx

**Supporting Information**: EJH_AML Consensus_Supplementary Results.docx

## Data Availability

All data relevant to the study are included in the article or uploaded as Supporting Information.
