# Correction to “A Delphi Consensus on Optimising the Care Pathway for Adult Patients With Acute Myeloid Leukaemia (AML): Strategies to Enhance Transplant Accessibility and Feasibility in the United Kingdom”

**DOI:** 10.1002/jha2.70379

**Published:** 2026-08-21

P. Mehta, F. A. M. Kinsella, T. P. Coats, A. B. Khan, A.‐L. Latif, and V. T. Potter, “A Delphi Consensus on Optimising the Care Pathway for Adult Patients With Acute Myeloid Leukaemia (AML): Strategies to Enhance Transplant Accessibility and Feasibility in the United Kingdom.” *eJHaem* 7, no. 4 (2026): e70353. https://doi.org/10.1002/jha2.70353

A visual/graphical abstract was previously missing and has been added to the online version of the original published article.

In the results section, the statements in Table 1 were wrongly formatted and should have been left‐aligned. Table 1 should display as shown below.

**TABLE 1:** Defined consensus statements and corresponding levels of agreement. All values are rounded to the nearest number.

**No**.	**Statement**	**Strongly agree**	**Tend to agree**	**Tend to disagree**	**Strongly disagree**	**Agreement score** **^a^** **(*n* = 75)**
**Topic 1. Early transplant discussion**						
**1**.	All patients diagnosed with acute myeloid leukaemia (AML) and eligible for curative treatment should be informed at diagnosis that stem cell transplantation may be part of their treatment pathway	80%	19%	1%	0%	**99%**
**2**.	All patients diagnosed with AML who have the potential for curative treatment (excluding acute promyelocytic leukaemia [APL]) should undergo human leukocyte antigen (HLA) typing at diagnosis to facilitate timely donor identification	80%	17%	1%	1%	**97%**
**3**.	Donor searches should be initiated as early as possible and suitable related donor options should be explored promptly with the patient at diagnosis	72%	25%	1%	1%	**97%**
**4**.	Transplant centres should be involved in the donor search to allow prompt initiation of an unrelated donor search should no suitable related donor is identified	69%	27%	3%	1%	**96%**
**5**.	There is lack of evidence supporting an age cut‐off for suitable sibling donors	17%	64%	19%	0%	**81%**
**6**.	To speed up donor workup, an age cutoff for sibling donors should be set by transplant centres to make pathways for referring hospitals clearer	39%	51%	9%	1%	**89%**
**7**.	All patients diagnosed with AML who have the potential for curative treatment (excluding APL) should be referred to a transplant specialist as soon as possible, within their first treatment cycle and molecular results sent as they become available	64%	31%	5%	0%	**95%**
**8**.	Measurable residual disease (MRD) markers should be identified at diagnosis, and subsequently monitored in accordance with ELN recommendations to support transplant decision‐making	77%	23%	0%	0%	**100%**
**Topic 2. Patient suitability**						
**9**.	Transplant recommendations should balance the risk of disease progression without transplant against the risks and toxicity associated with transplantation	72%	25%	3%	0%	**97%**
**10**.	All patients should undergo a comprehensive assessment of performance status, comorbidities and organ function, such as Haematopoietic Cell Transplantation‐specific Comorbidity Index (HCT‐CI) scoring, to evaluate their transplant risk	85%	15%	0%	0%	**100%**
**11**.	Fitness for transplant should be assessed holistically, including evaluation by a clinical psychologist, physiotherapist and dietitian as part of the multidisciplinary team (MDT)	60%	39%	1%	0%	**99%**
**12**.	Age alone should not be a barrier to transplant	53%	39%	8%	0%	**92%**
**13**.	Patients over 60 should consider undergoing a formal geriatric assessment	45%	41%	13%	0%	**87%**
**14**.	To improve standardisation of care and referral consistency, there should be strict disease criteria for transplant indications, as there are for other Cancer drug fund/NICE approved treatments, for example, *TP53* mutated disease, MRD‐positive disease, transplant in partial remission	59%	35%	7%	0%	**93%**
**15**.	Patients with primary refractory AML should be considered for transplant	64%	31%	5%	0%	**95%**
**16**.	Patient fitness for transplant should be reassessed after each cycle of chemotherapy	65%	31%	4%	0%	**96%**
**17**.	MRD positivity should not be a barrier to transplant but the patient may require additional therapy, especially if there is a time delay in securing a donor/transplant slot	44%	55%	1%	0%	**99%**
**18**.	MRD‐positive patients should undergo a higher intensity conditioning, such as myeloablative conditioning, before transplant if possible	37%	56%	5%	1%	**93%**
**Topic 3. Time to transplant**						
**19**.	Transplant delays can be reduced through early referral to a transplant centre	73%	25%	1%	0%	**99%**
**20**.	Early donor search and HLA typing help to prevent transplant delays	79%	20%	1%	0%	**99%**
**21**.	All eligible patients in first complete remission (CR1) should proceed to transplant as soon as adequate disease control is achieved and a suitable donor is available	68%	25%	7%	0%	**93%**
**22**.	All eligible patients in second complete remission (CR2) should proceed to transplant as soon as adequate disease control is achieved and a suitable donor is available, if they did not have a transplant in CR1	69%	28%	3%	0%	**97%**
**23**.	Patients should have comorbidities optimally managed during induction therapy to minimise transplant‐related risks	72%	27%	1%	0%	**99%**
**24**.	Additional chemotherapy cycles before transplant should be minimised once a patient has achieved CR1 unless necessary for disease control or relapse prevention	56%	36%	8%	0%	**92%**
**25**.	If additional chemotherapy cycles are administered after adequate disease control but before transplant, the MDT should investigate the reasons why this happened	45%	47%	8%	0%	**92%**
**Topic 4. The role of MDT assessment in transplant referral**						
**26**.	All AML patients for whom transplant is an option should be discussed in a transplant MDT	79%	20%	1%	0%	**99%**
**27**.	MDT discussions should include at least two clinicians with a special interest in AML, and should also involve input from a transplant specialist or transplant centre; otherwise, the patient should be referred to a regional AML MDT or an AML/transplant specialist at another centre	79%	20%	1%	0%	**99%**
**28**.	All AML clinicians should have access to a regional AML MDT to discuss complex patient cases	88%	12%	0%	0%	**100%**
**29**.	The reasons behind decisions to refer or not refer an AML patient for transplant should be formally documented at diagnosis during an MDT meeting	80%	19%	1%	0%	**99%**
**30**.	All transplant‐eligible AML patients should be discussed at an MDT meeting and with the transplant team when an integrated genetic (Haematological Malignancy Diagnostic Service, HMDS) report becomes available	72%	27%	1%	0%	**99%**
**31**.	A transplant centre should provide input into patient management during MDT discussions	72%	27%	1%	0%	**99%**
**Topic 5. Standardising the referral process and communication between centres**						
**32**.	Every referring hospital should have a designated transplant centre for AML referrals and discussions	80%	20%	0%	0%	**100%**
**33**.	Transplant centres should provide clear, standardised referral guidelines and processes for referring hospitals	79%	20%	1%	0%	**99%**
**34**.	MDT proformas should be standardised to ensure consistency and clarity in treatment decisions	80%	19%	1%	0%	**99%**
**35**.	Transplant centres and referring hospitals should establish clear communication protocols, including designated key contacts (e.g., clinical nurse specialists [CNS]) and a shared email address for the transplant centre	73%	25%	1%	0%	**99%**
**36**.	Efforts should be made to develop an electronic platform to enhance communication between transplant centres and referring hospitals	71%	28%	1%	0%	**99%**
**37**.	All transplant eligible patients should receive written information about the transplant process at diagnosis	55%	33%	12%	0%	**88%**
**38**.	All transplant eligible patients should receive written information about the transplant process when they achieve CR1 after first cycle of chemotherapy	63%	32%	5%	0%	**95%**
**39**.	The transplant and treating centres should agree on key patient outcomes that must be shared to ensure both teams are fully informed prior to the transplant	65%	31%	4%	0%	**96%**
**40**.	After each treatment cycle, disease reassessment, fitness reassessment and adverse events should be communicated to the transplant centre	67%	29%	4%	0%	**96%**
**41**.	Transplant centres should provide provisional transplant dates to the referring team to allow timely treatment and testing	68%	29%	3%	0%	**97%**
**42**.	Transplant decisions must be clearly communicated to the referring team by the transplant centre	84%	15%	1%	0%	**99%**
**43**.	Predicted outcomes, including transplant‐related mortality (TRM), overall survival and relapse risk, should be clearly communicated to the referring team and the patient (if the patient consents)	77%	21%	1%	0%	**99%**
**Topic 6. The role of shared care**						
**44**.	The transplant centre should define the post‐transplant follow‐up schedule before the transplant, including the frequency and location of reviews and monitoring	64%	33%	3%	0%	**97%**
**45**.	Specific shared care responsibilities, such as local blood tests, transfusion support and management of complications, should be agreed upon between the transplant centre and referring hospitals before transplantation	71%	28%	1%	0%	**99%**
**46**.	The agreed shared care plan, including responsibilities and follow‐up schedules, should be clearly explained to the patient and carers before transplantation	81%	17%	1%	0%	**99%**
**47**.	Shared care forms should include detailed patient clinical information and be communicated to local blood banks	72%	25%	3%	0%	**97%**

^a^ Percentages were rounded to the nearest whole number prior to assessing whether the threshold for consensus was met.

We apologize for these errors.

