# Supplementary material for: A Delphi Consensus on Optimising the Care Pathway for Adult Patients With Acute Myeloid Leukaemia (AML): Strategies to Enhance Transplant Accessibility and Feasibility in the United Kingdom
Source: EJHaem. 2026 Jul 13;7(4):e70353. doi: 10.1002/jha2.70353 (PMC13359021; doi:10.1002/jha2.70353)
Supplement: Supplementary file 1 — Supporting Information: EJH_AML Consensus_Supplementary Clinical Checklist.docx [file JHA2-7-e70353-s002.docx]

**SUPPLEMENTARY INFORMATION – CLINICAL CHECKLIST**

Checklist for the Diagnosis, Assessment, and Referral to Transplant of Patients with AML

| Step | ✓ | Comment/Note |
| --- | --- | --- |
| At Diagnosis (For Referral Centre) | | |
| AML diagnosis and eligibility for curative intent confirmed (exclude APL) | ☐ |  |
| Eligible patient has been informed that stem cell transplantation may be part of treatment | ☐ |  |
| Patient has been directed to resources on treatment choices and AML support groups | ☐ |  |
| Transplant Centre has been notified of the patient and donor discussions | ☐ |  |
| HLA typing has been initiated | ☐ |  |
| Donor search has been initiated (including related and unrelated donors) | ☐ |  |
| Local sibling donor age policy has been confirmed with the transplant centre | ☐ |  |
| Molecular/genetic results have been shared with the transplant centre | ☐ |  |
| MDT Assessments (For Referral Centre) | | |
| AML patient has been discussed in a Local AML MDT | ☐ |  |
| If complex case: AML patient has been discussed at Regional AML MDT | ☐ |  |
| MDT included ≥2 AML specialists | ☐ |  |
| MDT included transplant input | ☐ |  |
| MDT discussion of the patient included discussion of an integrated HMDS report | ☐ |  |
| MDT rationale for transplant decision has been formally documented | ☐ |  |
| If considered eligible for transplant, the patient has been provided with preliminary transplant information | ☐ |  |
| Transplant Eligibility considerations (For MDT Consideration) | | |
| Comprehensive patient assessment including HCT-CI score and organ function tests have been conducted | ☐ |  |
| Holistic assessments have been conducted as appropriate (psychology, dietetics, physiotherapy) | ☐ |  |
| If patient is ≥60: geriatric assessment has been considered | ☐ |  |
| Consider Patient related Factors:  Age  Performance Status  Comorbidities  Organ Function | ☐  ☐  ☐  ☐ |  |
| Consider Disease Related Factors:  TP53 Mutation  MRD Positivity  Remission Status  Refractory Disease | ☐  ☐  ☐  ☐ |  |
| The balance of transplant risks vs disease progression risk has been considered | ☐ |  |
| Treatment and Transplant Considerations (For Referral and Transplant Centres) | | |
| Nominated transplant centre for the referral has been confirmed | ☐ |  |
| Decision regarding patient eligibility for transplant has been confirmed with the transplant centre | ☐ |  |
| Decision regarding patient eligibility for transplant has been communicated to the patient | ☐ |  |
| Key clinical data to be shared throughout treatment has been agreed between referral centre and transplant centre | ☐ |  |
| Preliminary date for transplant has been received by referral centre from the transplant centre | ☐ |  |
| Detailed transplant information has been communicated to the referral centre and the patient by the transplant centre, including: | ☐ |  |
| Predicted Outcomes shared with Referral Centre and Patient:  Treatment Related Mortality  Overall Survival  Relapse Risk | ☐  ☐  ☐ |  |
| Patient fitness has been re-assessed after every treatment cycle | ☐ |  |
| Disease status, patient fitness and toxicities have been communicated to the transplant centre after every treatment cycle | ☐ |  |
| Post-transplant Follow-up (For Transplant Centre) | | |
| Transplant Centre has outlined post-transplant follow-up frequency and responsibilities | ☐ |  |
| Patient and/or carer have been informed of follow-up responsibilities and follow-up plan | ☐ |  |
| All forms and full clinical data have been shared with the transplant centre | ☐ |  |
| General Communication Considerations (For Referral and Transplant Centres) | | |
| Standardised referral guidelines are in place | ☐ |  |
| Standardised referral pro formas are in place | ☐ |  |
| Designated communication routes (shared email/key contact/CNS liaison) have been agreed | ☐ |  |
| Designated communication routes (shared email/key contact/CNS liaison) have been shared with the patient | ☐ |  |
