# Supplementary material for: A Delphi Consensus on Optimising the Care Pathway for Adult Patients With Acute Myeloid Leukaemia (AML): Strategies to Enhance Transplant Accessibility and Feasibility in the United Kingdom
Source: EJHaem. 2026 Jul 13;7(4):e70353. doi: 10.1002/jha2.70353 (PMC13359021; doi:10.1002/jha2.70353)
Supplement: Supplementary file 2 — Supporting Information: EJH_AML Consensus_Supplementary Results.docx [file JHA2-7-e70353-s001.docx]

**SUPPLEMENTARY INFORMATION – RESULTS**

**Figure S1.** Respondents by role.

**Figure S2.** Respondents by years of experience.

**Figure S3.** Respondents by institution.

**Figure S4.** Respondents by region and country.

**Table S1.** Variation of consensus scores by role. Subgroup and overall mean agreement percentages were rounded to the nearest whole number before assessing whether subgroup values differed by greater than ±10% from the overall mean.

| **No:** | **Statement:** | **Mean Score**  **n=75** |  | **Myeloid haematologist**  **n=34** | **General haematologist n=26** | **Stem cell transplanter n=15** |
| --- | --- | --- | --- | --- | --- | --- |
| **Topic 5. Standardising the referral process & communication between centres** | | | | | | |
| 42 | Transplant decisions must be clearly communicated to the referring team by the transplant centre | **99%** |  | 94% | 94% | 87% |
| 43 | Predicted outcomes, including Transplant-Related Mortality (TRM), overall survival, and relapse risk, should be clearly communicated to the referring team and the patient (if the patient consents) | **99%** |  | 97% | 97% | 80% |
| **Topic 6. The role of shared care** | | | | | | |
| 44 | The transplant centre should define the post-transplant follow-up schedule before the transplant, including the frequency and location of reviews and monitoring | **97%** |  | 97% | 97% | 73% |
| 45 | Specific shared care responsibilities, such as local blood tests, transfusion support, and management of complications, should be agreed on between the transplant centre and referring hospitals before transplantation | **99%** |  | 97% | 97% | 87% |
| 46 | The agreed shared care plan, including responsibilities and follow-up schedules, should be clearly explained to the patient and carers before transplantation | **99%** |  | 94% | 94% | 87% |

**Table S2.** Variation of consensus scores by years of experience. Subgroup and overall mean agreement percentages were rounded to the nearest whole number before assessing whether subgroup values differed by greater than ±10% from the overall mean.

| **No:** | **Statement:** | **Mean Score**  **n=75** |  | **Less than 5 years**  **n=3** | **5-10 years n=31** | **11-20 years n=31** | **More than 20 years n=10** |
| --- | --- | --- | --- | --- | --- | --- | --- |
| **Topic 1.**  **Early transplant discussion** | | | | | | | |
| 5 | There is lack of evidence supporting an age cut-off for suitable sibling donors | **81%** |  | 100% | 74% | 81% | 100% |
| 6 | To speed up donor work up, an age cut off for sibling donors should be set by transplant centres to make pathways for referring hospitals clearer | **89%** |  | 67% | 87% | 90% | 100% |
| **Topic 2.**  **Patient suitability** | | | | | | | |
| 13 | Patients over 60 should consider undergoing a formal geriatric assessment | **87%** |  | 100% | 81% | 90% | 90% |
| 14 | To improve standardisation of care and referral consistency, there should be strict disease criteria for transplant indications, as there are for other Cancer drug fund/NICE approved treatments, e.g., TP53 mutation disease, MRD positive disease, transplant in partial remission | **93%** |  | 67% | 90% | 97% | 100% |
| **Topic 5.**  **Standardising the referral process & communication between centres** | | | | | | | |
| 37 | All transplant eligible patients should receive written information about the transplant process at diagnosis | **88%** |  | 100% | 87% | 87% | 90% |
| **Topic 6.**  **The role of shared care** | | | | | | | |
| 44 | The transplant centre should define the post-transplant follow-up schedule before the transplant, including the frequency and location of reviews and monitoring | **97%** |  | 100% | 90% | 100% | 80% |
| 45 | Specific shared care responsibilities, such as local blood tests, transfusion support, and management of complications, should be agreed on between the transplant centre and referring hospitals before transplantation | **99%** |  | 100% | 94% | 100% | 80% |

**Table S3.** Variation of consensus scores by institution. Subgroup and overall mean agreement percentages were rounded to the nearest whole number before assessing whether subgroup values differed by greater than ±10% from the overall mean.

| **No:** | **Statement:** | **Mean Score**  **n=75** |  | **Transplant centre**  **n=39** | **Hospital providing intensive AML treatment n=30** | **Hospital providing supportive or low-intensity AML care only n=16** |
| --- | --- | --- | --- | --- | --- | --- |
| **Topic 1.**  **Early transplant discussion** | | | | | | |
| 3 | Donor searches should be initiated as early as possible and suitable related donor options should be explored promptly with the patient at diagnosis | **97%** |  | 100% | 97% | 83% |
| 6 | To speed up donor work up, an age cut off for sibling donors should be set by transplant centres to make pathways for referring hospitals clearer | **89%** |  | 87% | 90% | 100% |
| **Topic 2.**  **Patient suitability** | | | | | | |
| 12 | Age alone should not be a barrier to transplant | **92%** |  | 95% | 93% | 67% |
| **Topic 5.**  **Standardising the referral process & communication between centres** | | | | | | |
| 38 | All transplant eligible patients should receive written information about the transplant process when they achieve CR1 after first cycle of chemotherapy | **95%** |  | 97% | 93% | 83% |

**Table S4.** Variation of consensus scores by region. Subgroup and overall mean agreement percentages were rounded to the nearest whole number before assessing whether subgroup values differed by greater than ±10% from the overall mean.

| **No:** | **Statement:** | **Mean Score**  **n=75** |  | **London**  **n=19** | **Midlands**  **n=15** | **South West**  **n=9** | **South East**  **n=8** | **East of England**  **n=8** | **North West**  **n=8** | **North East and Yorkshire**  **n=4** | **Scotland**  **n=4** |
| --- | --- | --- | --- | --- | --- | --- | --- | --- | --- | --- | --- |
| **Topic 1.**  **Early transplant discussion** | | | | | | | | | | | |
| 2 | All patients diagnosed with AML who have the potential for curative treatment (excluding Acute Promyelocytic Leukaemia (APL)) should undergo Human Leukocyte Antigen (HLA) typing at diagnosis to facilitate timely donor identification | **97%** |  | 100% | 93% | 100% | 100% | 100% | 100% | 100% | 75% |
| 3 | Donor searches should be initiated as early as possible and suitable related donor options should be explored promptly with the patient at diagnosis | **97%** |  | 100% | 100% | 100% | 100% | 100% | 88% | 100% | 75% |
| 4 | Transplant centres should be involved in the donor search to allow prompt initiation of an unrelated donor search should no suitable related donor is identified | **96%** |  | 89% | 100% | 100% | 100% | 100% | 100% | 100% | 75% |
| 5 | There is lack of evidence supporting an age cut-off for suitable sibling donors | **81%** |  | 95% | 60% | 89% | 63% | 75% | 100% | 75% | 100% |
| 6 | To speed up donor work up, an age cut off for sibling donors should be set by transplant centres to make pathways for referring hospitals clearer | **89%** |  | 84% | 100% | 100% | 88% | 100% | 100% | 50% | 50% |
| 7 | All patients diagnosed with AML who have the potential for curative treatment (excluding APL) should be referred to a transplant specialist as soon as possible, within their first treatment cycle and molecular results sent as they become available | **95%** |  | 100% | 100% | 89% | 88% | 100% | 88% | 100% | 75% |
| **Topic 2.**  **Patient suitability** | | | | | | | | | | | |
| 12 | Age alone should not be a barrier to transplant | **92%** |  | 100% | 87% | 100% | 75% | 88% | 100% | 100% | 75% |
| 13 | Patients over 60 should consider undergoing a formal geriatric assessment | **87%** |  | 84% | 93% | 100% | 88% | 88% | 88% | 75% | 50% |
| 14 | To improve standardisation of care and referral consistency, there should be strict disease criteria for transplant indications, as there are for other Cancer drug fund/NICE approved treatments, e.g., TP53 mutation disease, MRD positive disease, transplant in partial remission | **93%** |  | 89% | 100% | 100% | 88% | 88% | 100% | 75% | 100% |
| 18 | MRD-positive patients should undergo a higher intensity conditioning, such as myeloablative conditioning, before transplant if possible | **93%** |  | 95% | 100% | 100% | 88% | 88% | 100% | 75% | 75% |
| **Topic 3. Time to transplant** | | | | | | | | | | | |
| 19 | Transplant delays can be reduced through early referral to a transplant centre | **99%** |  | 100% | 100% | 100% | 100% | 88% | 100% | 100% | 100% |
| 21 | All eligible patients in first complete remission (CR1) should proceed to transplant as soon as adequate disease control is achieved and a suitable donor is available | **93%** |  | 95% | 87% | 100% | 88% | 100% | 100% | 100% | 75% |
| 24 | Additional chemotherapy cycles before transplant should be minimised once a patient has achieved CR1 unless necessary for disease control or relapse prevention | **92%** |  | 89% | 100% | 89% | 88% | 100% | 100% | 75% | 75% |
| 25 | If additional chemotherapy cycles are administered after adequate disease control but before transplant, the MDT should investigate the reasons why this happened | **92%** |  | 95% | 87% | 100% | 88% | 100% | 88% | 100% | 75% |
| **Topic 4. The role of MDT assessment in transplant referral** | | | | | | | | | | | |
| 30 | All transplant eligible AML patients should be discussed at an MDT meeting and with the transplant team when an integrated genetic (Haematological Malignancy Diagnostic Service, HMDS) report becomes available | **99%** |  | 100% | 100% | 100% | 88% | 100% | 100% | 100% | 100% |
| **Topic 5.**  **Standardising the referral process & communication between centres** | | | | | | | | | | | |
| 35 | Transplant centres and referring hospitals should establish clear communication protocols, including designated key contacts (e.g., Clinical Nurse Specialists (CNS)) and a shared email address for the transplant centre | **99%** |  | 100% | 100% | 100% | 100% | 100% | 100% | 75% | 100% |
| 37 | All transplant eligible patients should receive written information about the transplant process at diagnosis | **88%** |  | 89% | 87% | 78% | 88% | 88% | 88% | 100% | 100% |
| 38 | All transplant eligible patients should receive written information about the transplant process when they achieve CR1 after first cycle of chemotherapy | **95%** |  | 95% | 80% | 100% | 100% | 100% | 100% | 100% | 100% |
| 39 | The transplant and treating centres should agree on key patient outcomes that must be shared to ensure both teams are fully informed prior to the transplant | **96%** |  | 95% | 100% | 100% | 75% | 100% | 100% | 100% | 100% |
| 40 | After each treatment cycle, disease reassessment, fitness reassessment and adverse events should be communicated to the transplant centre | **96%** |  | 89% | 100% | 100% | 100% | 100% | 100% | 75% | 100% |
| 41 | Transplant centres should provide provisional transplant dates to the referring team to allow timely treatment and testing | **97%** |  | 89% | 93% | 89% | 100% | 100% | 100% | 100% | 75% |
| 42 | Transplant decisions must be clearly communicated to the referring team by the transplant centre | **99%** |  | 89% | 93% | 100% | 100% | 100% | 100% | 100% | 75% |
| 43 | Predicted outcomes, including Transplant-Related Mortality (TRM), overall survival, and relapse risk, should be clearly communicated to the referring team and the patient (if the patient consents) | **99%** |  | 95% | 93% | 89% | 100% | 100% | 100% | 100% | 75% |
| **Topic 6.**  **The role of shared care** | | | | | | | | | | | |
| 44 | The transplant centre should define the post-transplant follow-up schedule before the transplant, including the frequency and location of reviews and monitoring | **97%** |  | 89% | 93% | 100% | 100% | 88% | 100% | 100% | 75% |
| 45 | Specific shared care responsibilities, such as local blood tests, transfusion support, and management of complications, should be agreed on between the transplant centre and referring hospitals before transplantation | **99%** |  | 95% | 93% | 100% | 88% | 100% | 100% | 100% | 75% |
| 46 | The agreed shared care plan, including responsibilities and follow-up schedules, should be clearly explained to the patient and carers before transplantation | **99%** |  | 89% | 93% | 100% | 100% | 100% | 100% | 100% | 75% |
| 47 | Shared care forms should include detailed patient clinical information and be communicated to local blood banks | **97%** |  | 89% | 93% | 100% | 100% | 88% | 100% | 100% | 75% |
